# Efficacy of Seed Cycling as an Integrative Therapy for Premenstrual Syndrome and Polycystic Ovary Syndrome in Reproductive-Aged Women: A Systematic Review

**DOI:** 10.7759/cureus.90997

**Published:** 2025-08-25

**Authors:** Divyashri R Nagarajan, Daya Mani Jacob, Manal Munir Mufti, Smiruti Rajesh, Rajani Dube

**Affiliations:** 1 Obstetrics and Gynaecology, Burjeel Medical City, Abu Dhabi, ARE; 2 Internal Medicine, Burjeel Medical City, Abu Dhabi, ARE; 3 Obstetrics and Gynaecology, Emirates Health Services, Abu Dhabi, ARE; 4 Nutrition, South Warwickshire NHS Foundation Trust, Warwick, GBR; 5 Obstetrics and Gynaecology, Ras Al Khaimah Medical and Health Sciences University, Ras al Khaimah, ARE

**Keywords:** hormonal imbalance, menstrual irregularities, polycystic ovary syndrome, premenstrual syndrome, seed cycling, women's health

## Abstract

Premenstrual syndrome (PMS) and polycystic ovary syndrome (PCOS) are common hormonal disorders in reproductive-aged women. Conventional treatments often have side effects, highlighting the need for natural alternatives such as seed cycling, a dietary strategy involving flax, pumpkin, sesame, and sunflower seeds consumed in specific phases of the menstrual cycle. This systematic review evaluated studies from 2015 to 2025 (PubMed, Scopus) on seed cycling or its components in women aged 15-49 years with PMS or PCOS diagnosed using standardized criteria. Eligible designs included randomized controlled trials, cohort, case-control, observational, quasi-experimental, and case series. Non-human studies, reviews, opinion pieces, conference abstracts, and studies outside the age range or before 2015 were excluded. The PRISMA 2020 guidelines were followed, and the Quality Assessment with Diverse Studies Tool assessed risk of bias. Primary outcomes included changes in hormonal markers (e.g., estrogen, progesterone, LH/FSH ratio, testosterone), symptom scores for PMS, and metabolic parameters (e.g., BMI, lipid profile, insulin resistance). Ten studies (n = 635) with low to medium risk of bias reported that seed cycling, particularly flax and sesame seeds, was associated with improved menstrual regularity, reduced PMS symptom severity, favorable modulation of sex hormone levels, and improvements in metabolic profiles. While findings suggest that seed cycling is a low-cost, safe, and potentially effective adjunct for managing PMS and PCOS, the conclusion is based on small sample sizes and moderate-quality evidence, underscoring the need for larger randomized controlled trials and standardized intervention protocols.

## Introduction and background

Premenstrual syndrome (PMS) and polycystic ovary syndrome (PCOS) are two of the most common reproductive health disorders that have a significant impact on the health and quality of life of women of reproductive age. PMS is defined by cyclical emotional and physical symptoms that occur before menstruation and resolve shortly after its onset, while PCOS is a complex endocrine disorder characterized by irregular ovulation, elevated androgen levels, and polycystic ovarian morphology [1]. Globally, PCOS affects approximately 7-10% of women of reproductive age, while PMS is experienced to varying degrees by the majority of menstruating women [2,3]. These conditions not only cause physical discomfort but also impair work productivity, emotional well-being, and social participation, highlighting their significance beyond individual health.

Disruptions in the hypothalamic-pituitary-ovarian axis, insulin resistance, chronic inflammation, and obesity contribute significantly to PCOS pathogenesis, while cyclical fluctuations in estrogen and progesterone underlie PMS symptoms such as mood swings, fatigue, and bloating. Both conditions share hormonal imbalances that disrupt reproductive and metabolic function, with marked effects on quality of life, daily functioning, and mental health [1,4,5]. PCOS is diagnosed based on the Rotterdam criteria, requiring at least two of the following: oligo- or anovulation, clinical or biochemical signs of hyperandrogenism, and polycystic ovaries visible via ultrasound [6,7]. PMS is typically identified by symptom tracking over at least two menstrual cycles, with resolution at menstruation onset [1,8]. 

Although these conditions are prevalent and impactful, the underlying mechanisms remain incompletely understood, and current treatments often address symptoms rather than root causes. Nonsteroidal anti-inflammatory drugs (NSAIDs) and selective serotonin reuptake inhibitors (SSRIs) are commonly used to manage PMS, while hormonal contraceptives are the first-line pharmacologic intervention for PCOS to regularize menstruation and lower hyperandrogenic symptoms. Women with insulin resistance are often treated with metformin, and lifestyle interventions such as diet and exercise are recommended concurrently [1,4,9]. However, long-term use of hormonal contraceptives may not address the underlying endocrine abnormalities, metformin may cause gastrointestinal side effects, and antidepressants can lead to weight gain, fatigue, or sexual dysfunction [10,11]. 

These limitations have prompted interest in safe, accessible, and patient-centered integrative approaches that could complement or reduce reliance on conventional therapies. The biological rationale for seed cycling lies in the presence of bioactive compounds in specific seeds that may modulate hormonal pathways relevant to PMS and PCOS. Flaxseeds and sesame seeds contain lignans and phytoestrogens, which can influence estrogen metabolism by binding to estrogen receptors and modulating the conversion of stronger estrogens (estradiol) to weaker forms (estrone), potentially alleviating estrogen-dominant states seen in PMS and rebalancing estrogen-progesterone ratios in PCOS. Flaxseed lignans may also affect sex hormone-binding globulin (SHBG) levels and improve androgen metabolism, while sesame seed phytoestrogens may support luteal-phase estrogen balance. Pumpkin seeds are rich in zinc, which supports follicle-stimulating hormone (FSH) and luteinizing hormone (LH) synthesis, thereby promoting ovulation. Sunflower seeds, high in vitamin E and selenium, may enhance progesterone production and exert antioxidant effects, protecting ovarian and thyroid function. Collectively, these nutrients may influence key mechanisms implicated in PMS and PCOS, including gonadotropin release, ovarian steroidogenesis, insulin sensitivity, and oxidative stress [5,9,11].

**Figure 1:**
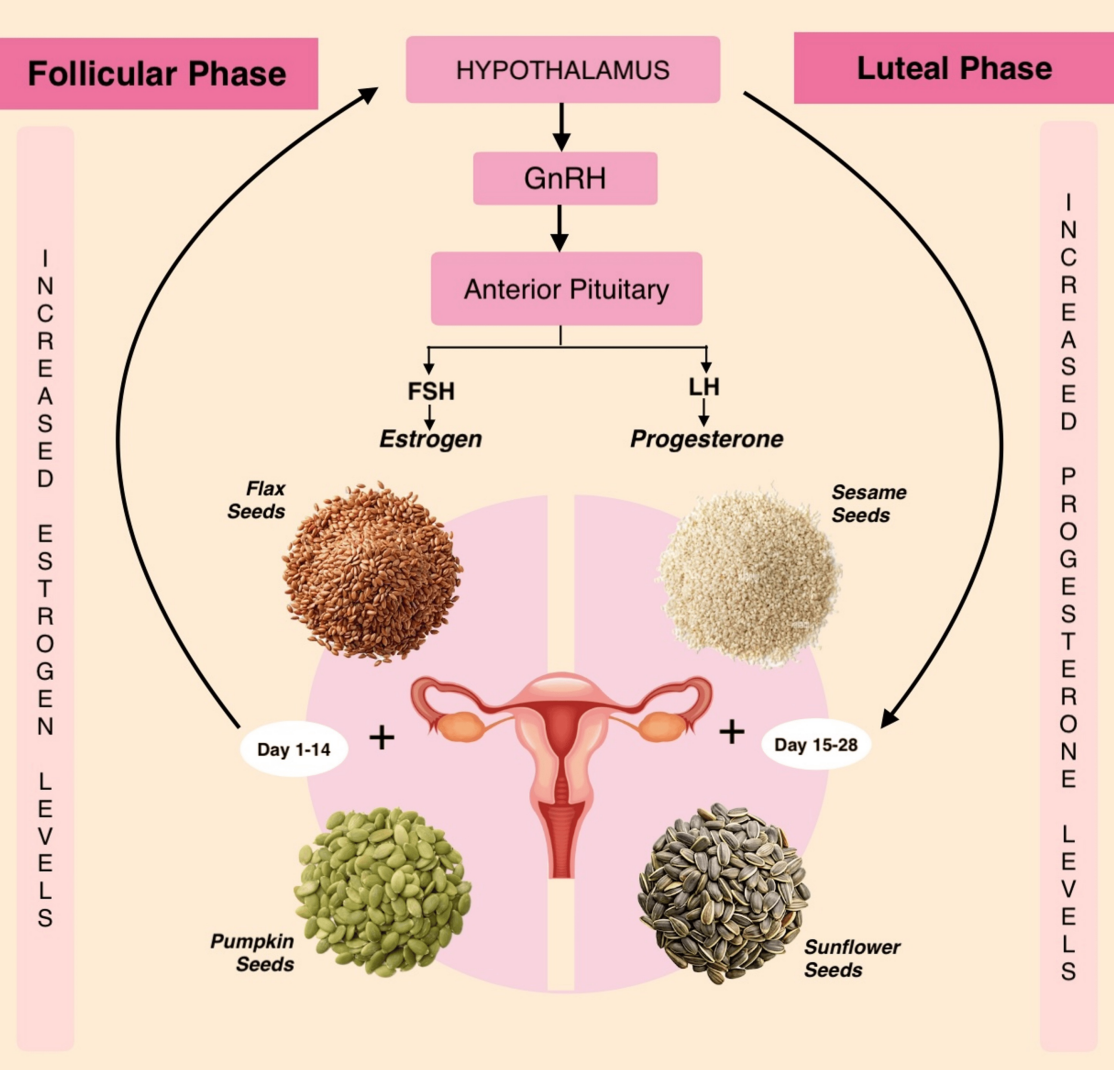
Phased Nutritional Intervention Through Seed Cycling in Reproductive-Age Women Adapted from Mahapatra et al. [11]

Seed cycling involves consuming flax and pumpkin seeds during the follicular phase (Days 1-14) to support estrogen metabolism and ovulation, and sesame and sunflower seeds during the luteal phase (Days 15-28) to enhance progesterone production and maintain hormonal balance (Figure 1). The cyclical combination of these nutrient profiles is hypothesized to synergistically modulate hormonal rhythms, potentially reducing PMS symptom severity and improving menstrual regularity in PCOS.

Despite its popularity in integrative health practices, seed cycling remains under-researched, with no comprehensive synthesis of its clinical evidence representing a clear gap this review seeks to address. The aim of this systematic review is to evaluate the effectiveness of seed cycling, a dietary approach using flax, pumpkin, sunflower, and sesame seeds in a cyclical pattern, on alleviating symptoms in reproductive-aged women with PMS and/or PCOS. Outcomes of interest include improvements in mood, cramps, mastalgia, cycle regularity, and other hormone-related symptoms, compared to placebo, standard care, or no intervention.

## Review

Methodology

This review was conducted in accordance with the Preferred Reporting Items for Systematic Reviews and Meta-Analyses (PRISMA) 2020 guidelines. A comprehensive literature search was performed between April 15, 2025, and May 15, 2025, using electronic databases including PubMed and Scopus. The search strategy combined Medical Subject Headings (MeSH) and free-text terms, using the string: ("seed cycling" OR "seed rotation" OR "flax seed" OR "pumpkin seed" OR "sunflower seed" OR "sesame seed") AND ("premenstrual syndrome" OR PMS OR "premenstrual symptoms" OR "menstrual symptoms" OR "polycystic ovary syndrome" OR PCOS OR "polycystic ovarian disease" OR PCOD). 

Inclusion criteria were English-language human studies published between 2015 and 2025, involving reproductive-aged women (15-49 years) diagnosed with PMS or PCOS using established criteria such as ACOG, Rotterdam, or DSM-5. Eligible designs included randomized controlled trials, cohort studies, case-control studies, observational studies, quasi-experimental trials, and case series. Interventions had to involve seed cycling or its components (flaxseed, pumpkin, sesame, sunflower), either alone or in combination with integrative therapies.

Exclusion criteria included theses, dissertations, unpublished work, conference abstracts, expert opinion, reviews, editorials, and studies in languages other than English where translations were unavailable. Non-human studies, postmenopausal women, adolescents under 15 years, studies published before 2015, and those lacking outcome measures related to menstrual health or hormonal parameters were excluded.

**Figure 2:**
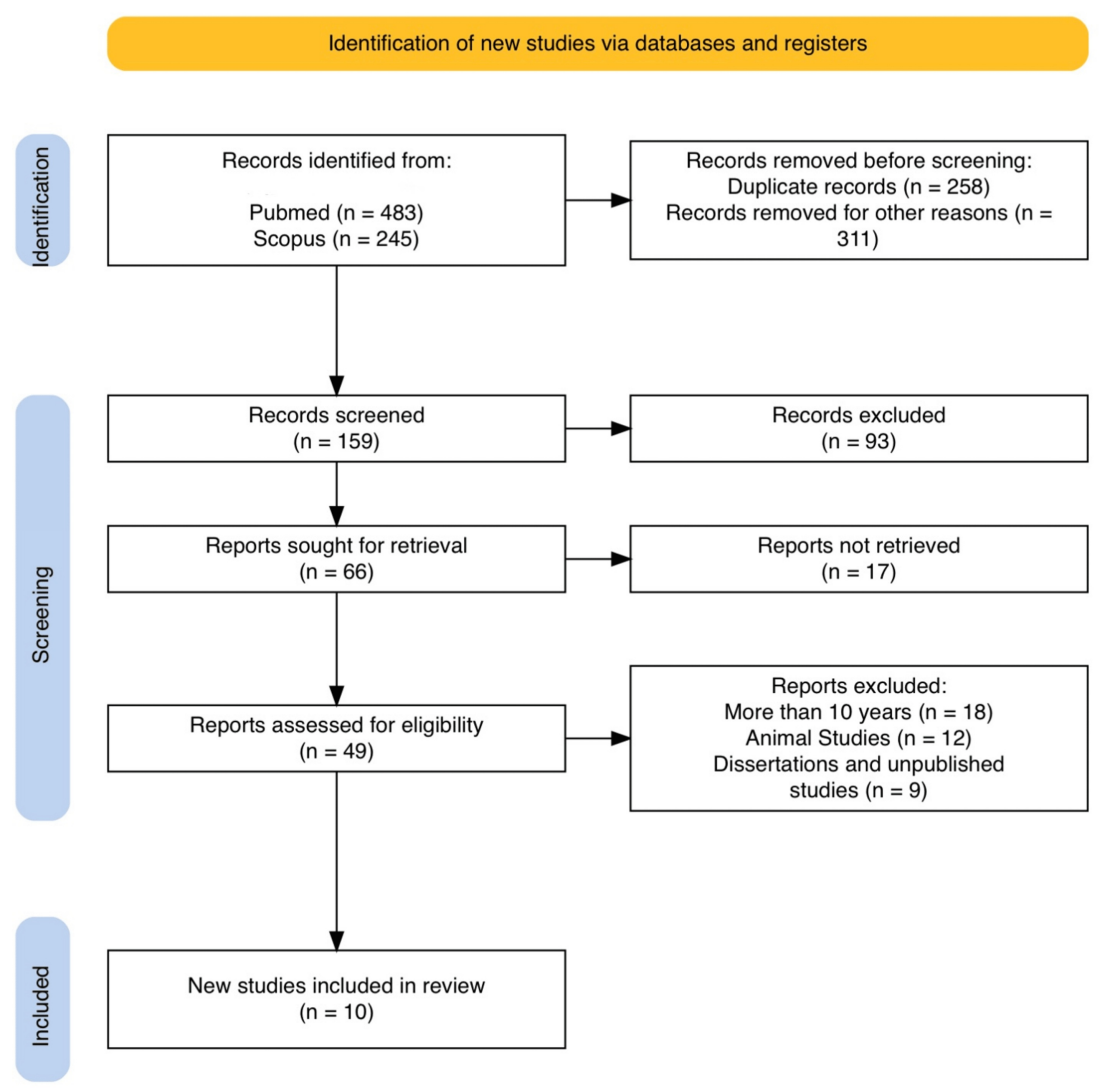
Flow diagram of study selection based on PRISMA 2020 guidelines Adapted from Haddaway et al. [12].

The study selection process adhered to PRISMA methodology. The initial search yielded 728 records (PubMed: 483, Scopus: 245). After removing 258 duplicates, 470 unique records remained. Screening excluded 311 studies for non-English language, irrelevant conditions, animal data, commentaries, conference proceedings, expert opinions, reviews, or interventions unrelated to seeds. This left 159 articles for title and abstract review, from which 93 were excluded for not meeting the inclusion criteria. Full texts were retrieved for 66 studies; 17 could not be accessed. Among the 49 full texts assessed, 39 were excluded, 18 were older than 10 years, 12 were animal studies, and 9 were dissertations or unpublished work. Ten studies met all criteria and were included in the final synthesis. The PRISMA flow diagram (Figure 2) outlines this process.

Quality assessment of the included studies was performed using the Quality Assessment for Diverse Studies (QuADS) tool [13], which evaluates methodological rigor across 13 criteria scored from 0 to 3, yielding a maximum of 39 points. Scores were converted to percentages and categorized as high quality (>75%), good quality (50-75%), moderate quality (25-50%), or poor quality (<25), and the results have been summarized in Table 1. Two independent reviewers performed the assessments, resolving disagreements through consensus and, when necessary, consulting a third reviewer. Inter-rater reliability was high (Cohen’s kappa = 0.82). Six studies were rated high quality, and four were rated good quality, with overall scores ranging from 51.3% to 97.4% (mean: 77.4%). No studies were rated low quality. Studies with a high risk of bias were excluded to ensure the reliability of the synthesized evidence.

**Table 1:** Quality appraisal of included studies using the QuADS tool > 75% (high), 50-75% (good), 25-50%(moderate), <25% (poor) QuADS: Quality Assessment for Diverse Studies

Study (Author, Year)	QuADS Score (/39)	Percentage (%)	Quality Rating
Farzana et al., 2015 [4]	29	74.36%	Good
Rasheed et al., 2023 [5]	33	84.60%	High
Ajith, 2024 [9]	30	76.90%	High
Haidari et al., 2020 [10]	38	97.40%	High
Kour et al., 2024 [14]	29	74.36%	Good
Dhamija et al., 2025 [15]	20	51.30%	Good
Yavari et al., 2016 [16]	37	94.90%	High
Mohammadian et al., 2015 [17]	31	79.50%	High
Matar et al., 2021 [18]	24	61.50%	Good
Najdgholami et al., 2025 [19]	37	94.90%	High

Outcomes assessed included PMS/PCOS symptoms, hormones, menstrual regularity, mood, and metabolic changes. Data from the included studies were tabulated using Microsoft Excel (Microsoft Corp., Redmond, WA) to summarize key characteristics, including study year, study design, country, age group, sample size, seed(s) used, and duration of intervention, and reported outcomes related to PMS and PCOS. Given the limited number and quality of eligible studies and the absence of uniform outcome metrics, a meta-analysis was not feasible. However, a qualitative synthesis was performed to identify patterns in efficacy, directions of effects, methodological strength, and limitations. 

Results

To evaluate the therapeutic potential of seed cycling and individual seed-based interventions in the management of PCOS and PMS, 10 eligible studies published between 2015 and 2025 were included. Interventions commonly involved dietary supplementation with flax, pumpkin, sunflower, and sesame seeds, administered either individually or as part of a seed cycling protocol, over periods ranging from 2-6 months. The studies collectively assessed outcomes such as menstrual regularity, hormonal balance, anthropometric indices, metabolic parameters, and symptom relief. Key characteristics and findings from each study are summarized in Table 2. 

**Table 2:** Literature Analysing the Efficacy of Seed Cycling in PMS and PCOS in Reproductive Age Women

Study, year	Study design	Country, region	Age group (in years)	Sample size (n)	Seed used	Duration	Key results
Farzana et al., 2015 [4]	Interventional Study	Tamil Nadu, India	18-35 with PCOS	32	Flaxseeds	3 months	Significant reductions in mean ovarian volume (right ovary –3.35 c.mm, p < 0.001; left ovary –2.383 c.mm, p < 0.001) and follicular count (right –4.259, p < 0.001; left –4.519, p < 0.001) were noted, while random blood sugar showed no significant change (p = 0.713). A total of 17 (56.7%) subjects did not have peripheral follicles, while 16 (53.3%) exhibited normal ovarian echogenicity. After flaxseed therapy, 10 (33.3%) experienced a regular menstrual cycle, 5 (16.7%) noted an increase in frequency, 9 (30%) showed no change, and 3 (10%) achieved pregnancy. There was no notable difference in hirsutism.
Rasheed et al., 2023 [5]	Interventional study	Pakistan	15-40 with PCOS	90	Flaxseed, pumpkin, sunflower, and sesame seeds	3 months	A portion-controlled diet and seed cycling reduced FSH levels by 1.2% to 2.5%. LH levels were reduced by 1.5%–2%
Ajith, 2024 [9]	Interventional study	Karnataka, India	20-32 with PCOS	30	Flaxseed, pumpkin, sunflower, and sesame seeds	3 months	Before therapy, only 6 (8.5%) women had a normal menstrual cycle. Following seed cycling therapy, 18 (78%) had regularization of their menstrual cycle, and 9 (19%) had improvement in the frequency.19(63.3%) subjects had hirsutism, and there was no improvement in hirsutism after treatment. There was no statistically significant difference in the random blood sugar level before and after treatment (p-value – 0.534)
Haidari et al., 2020 [10]	Randomized clinical trial	Iran	18–45 with PCOS	41	Flaxseed	12 weeks	Flaxseed supplementation in patients with PCOS could improve some biochemical and anthropometric markers, at least partially, through amelioration of dyslipidemia, obesity, IR, and inflammation. The results indicated a significant decrease in body weight (P = 0.001), waist circumference (P = 0.007), BMI (P = 0.001), insulin concentration (P = 0.021), Homeostatic Model Assessment of Insulin Resistance (P = 0.034), TG (P = 0.013), and leptin (P = 0.007) and a significant increase in HDL-C (P < 0.001) and adiponectin (P = 0.017) in the flaxseed group compared to the control group.
Kour et al., 2024 [14]	Cohort interventional study	Karnataka, India	18-40 with PCOS	290	Flaxseed, pumpkin, sunflower, and sesame seeds	3 months	Significant improvement in waist circumference, hip circumference, and waist-hip ratio (*p<0.05) was observed. At the end of 12 weeks, the intervention group had decreased FBS by 4.43%, total cholesterol by 5.49%, and LDL by 17.29%, and increased HDL by 19.29%, respectively. They also showed more substantial Improvements, with FSH levels improving by 41.61%, LH by 35.87%, prolactin by 13.37%, and testosterone by 3.42%. The energy levels were improved by 5.28%, carbohydrates by 12%, protein by 22.25%, and fats by 12.22% at the end of 12 weeks.
Dhamija et al., 2025 [15]	Case study	Maharashtra, India	29 with PCOS	1	Flaxseed, pumpkin, sunflower, and sesame seeds	6 months	After 6 months, the patient experienced notable improvements, such as regular menstrual cycles, a reduction in body mass index ranging from 29 to 24 kg/m², and normalized hormone levels.
Yavari et al., 2016 [16]	Single-blinded clinical trial	Iran	20-40 with PCOS	27	Sesame seeds	9 weeks	18 (72%) of the patients experienced bleeding after receiving the sesame. In the sesame group, 4 (50%) patients reported on-time menstruation for the next (drug-free) episodes compared to the progesterone group (p=0.016).
Mohammadian et al., 2015 [17]	Control trial	Iran	18-25 with PMS	24	Sesame Seeds	3 months	The pain of dysmenorrhea and the amount of medications consumed for PMS were statistically decreased in periods with the consumption of sesame.
Matar et al., 2021 [18]	Interventional trial	Egypt	18-40 women with PCOS	30	Flaxseed	3 months	Among the 10 women who were given flaxseeds, FSH increased most significantly in the flaxseed group, indicating better follicular development. LH and AMH decreased significantly in the flaxseed group compared to other groups, suggesting a reduction in hyperandrogenism and ovarian dysfunction. 4 women in the flaxseed group became pregnant, and most had regular menstrual cycles.
Najdgholami et al., 2025 [19]	Open-label randomized controlled clinical trial	Iran	20- 34.5 years with PCOS	70	Flaxseed	12 weeks	FSH levels showed a significant increase in the flaxseed group compared to the control group. FSH levels increased from 9.72 ± 11.95 µU/mL at baseline to 10.59 ± 12.14 µU/mL after the intervention (p = 0.027). Additionally, the LH/FSH ratio significantly decreased following flaxseed supplementation (p = 0.031)

Study Characteristics

A total of 10 studies, conducted between 2015 and 2025, were included in this review, comprising various interventional designs: five interventional trials, two randomized controlled trials, one cohort study, one single-blinded trial, and one case study. The studies originated predominantly from South Asia and the Middle East, including India (n=4), Iran (n=4), Pakistan (n=1), and Egypt (n=1). All participants were reproductive-aged women (range: 15-45 years) diagnosed with either PCOS or Premenstrual Syndrome (PMS). Sample sizes varied widely, from a single case to 290 participants. The most commonly used intervention was seed cycling, involving flax, pumpkin, sunflower, and sesame seeds, administered over 2-6 months. Several studies focused on flaxseed alone (n=4), while two assessed sesame seed (n=2)supplementation.

Menstrual Cycle Regulation

Menstrual regularity improved across several studies. Farzana et al. (2015) reported that 33.3% of women experienced regular cycles after flaxseed use, with 10% achieving pregnancy [4]. Ajith (2024) observed 78% menstrual cycle normalization following seed cycling [9]. In Matar et al. (2021), regular cycles were restored in most women, and four participants conceived after three months of flaxseed intake [18]. Dhamija et al. (2025) described complete menstrual normalization in a case report after six months of seed therapy [15]. Yavari et al. (2016) also found that 72% experienced withdrawal bleeding with sesame seeds and 50% resumed on-time menstruation (p=0.016) [16].

Hormonal Modulation

Hormonal changes were a consistent finding across studies. Rasheed et al. (2023) observed modest reductions in FSH (1.2-2.5%) and LH (1.5-2%) [5]. Kour et al. (2024) found significant increases in FSH (41.61%), LH (35.87%), prolactin (13.37%), and testosterone (3.42%) [14]. Najdgholami et al. (2025) reported a statistically significant increase in FSH (p = 0.027) and a decrease in LH/FSH ratio (p = 0.031) following flaxseed supplementation [19]. Similarly, Matar et al. (2021) noted a significant increase in FSH and decreases in LH and AMH, indicating improved follicular function and reduced ovarian dysfunction [18].

Metabolic and Anthropometric Improvements

Improvements in metabolic markers were documented in five studies. Haidari et al. (2020) found flaxseed reduced BMI (p = 0.001), waist circumference, insulin, HOMA-IR, triglycerides, and leptin, while increasing HDL-C (p < 0.001) and adiponectin [10]. Kour et al. (2024) reported reductions in fasting blood sugar (−4.43%), LDL (−17.29%), and increases in HDL (19.29%), along with favorable changes in waist-to-hip ratio and macronutrient intake. Dhamija et al. (2025) reported a BMI reduction from 29 to 24 kg/m² after six months [14,15].

Potential Benefits in Women’s Reproductive Health

Seed-based interventions, such as sesame and flaxseed supplementation, have shown promising results in women’s reproductive health. Research by Mohammadian et al. (2015) demonstrated that sesame seed supplementation significantly alleviated dysmenorrhea and reduced the need for medication among women with premenstrual syndrome (PMS) [17]. Additionally, studies by Farzana et al. (2015) and Matar et al. (2021) reported improved fertility outcomes, with pregnancy occurring in 10% and 40% of women receiving flaxseed supplementation, respectively [4,18]. Importantly, no adverse effects were reported across the included studies, although data on long-term safety remain limited.

These collective findings are detailed in Table 3, which presents findings from the included studies, outlining reported outcomes, statistical significance, and key methodological considerations to contextualize the current evidence on seed cycling in PMS and PCOS.

**Table 3:** Statistical Summary of Findings The table summarizes the outcomes reported across included studies, indicating the number of studies addressing each outcome, the general direction of effects observed (improvement, no change, or mixed results), and the presence or absence of statistical significance as reported (p < 0.05 denotes significance; NS means not significant; NR indicates that statistical testing was not reported). The notes section highlights key limitations such as heterogeneity in outcome definitions, measurement methods, and sample sizes, which may affect the interpretation and comparability of results across studies.

Outcome	Number of Studies	Effect Direction	Statistical Significance	Notes / Limitations
Menstrual regularity	5	Improvement in 3 studies; Mixed results in 2	p<0.05 in 2 studies; Not significant (NS) in 1; Not reported (NR) in 2	Outcome definitions varied; follow-up durations inconsistent
PMS symptom severity	6	Improvement in 4 studies; No change in 2	p<0.05 in 3 studies; NR in 3	Varied assessment tools (e.g., symptom questionnaires, diaries); heterogeneity limits pooling
Hormonal profile (estrogen, progesterone)	4	Progesterone increased in 2 studies; No estrogen change in 3	p<0.05 in 1 study; NR in others	Timing of hormone testing and assay methods differed across studies
PCOS symptom improvement (acne, hirsutism)	3	Improvement in 2 studies; Mixed results in 1	p<0.05 in 1 study; NR in others	Diagnostic criteria inconsistent; small sample sizes limit conclusions
Adverse effects	2	None reported	N/A	Reporting sparse; possible underreporting

Discussion

Seed cycling may be effective because of the specific nutrients provided by the seeds consumed during each phase of the menstrual cycle. According to the findings in the table, seed cycling may be a useful non-pharmacological remedy for the symptoms and irregularities associated with menstruation. However, it is challenging to conclude that seed cycling alone was responsible for these improvements due to the small sample size and absence of a control group. Additionally, it is important to note that most of the studies used self-reported data, which can be prone to social desirability bias and recall bias [10]. Interpretation of the pooled findings is limited by heterogeneity in outcome definitions and metrics, with some studies assessing menstrual regularity via self-report and others using cycle length tracking, and by inconsistent statistical reporting.

Plant phytoestrogenic substances called lignans, which are abundant in flaxseeds, have the ability to affect the metabolism of estrogen. In a randomized open-label controlled clinical trial by Haidari et al. (2020) [10] investigated the effects of flaxseed supplementation on metabolic and hormonal parameters in 41 women with PCOS. Over 12 weeks, participants in the intervention group received 30 g/day of ground flaxseed. The results showed significant improvements in insulin sensitivity, total testosterone, and hirsutism scores in the flaxseed group compared to controls. Additionally, menstrual regularity improved notably in the intervention group. These findings suggest that flaxseed may be beneficial in managing hormonal imbalances and metabolic disturbances in women with PCOS. However, little is known about the precise biological processes by which lignans influence hormone regulation at the molecular level [10]. Lignans, key dietary phytoestrogens, are metabolized by gut microbiota into enterolignans (e.g., enterodiol, enterolactone), which interact with estrogen receptors (ERα/ERβ) to exert weak estrogenic or anti-estrogenic effects depending on tissue context and concentration. Recent mechanistic studies demonstrate that lignans act as selective estrogen receptor modulators (SERMs), binding to estrogen receptors and thereby modulating gene expression involved in steroidogenesis and hormone metabolism [20]. This receptor interaction may explain their capacity to restore hormonal balance in endocrine disorders like PCOS. Musazadeh et al.'s systematic review and meta-analysis showed that flaxseed supplementation has an impact on hormones and can alter the profile of sex hormones in adults [21,22]. 

Zinc, abundant in pumpkin seeds, is a crucial cofactor for numerous enzymes and transcription factors involved in the synthesis of reproductive hormones. It plays a role in the pituitary release of gonadotropins (FSH and LH), which are essential for follicular maturation and ovulation. Beyond this, zinc influences ovarian development, oocyte maturation, and early embryonic development, regulating meiotic progression via the MOS-MAPK pathway and triggering the “zinc spark” at fertilization [23].

Reduced menstrual discomfort and better menstrual health are correlated with adequate zinc levels. Zinc supports reproductive health, according to studies like Syed et al. 2019. However, studies evaluating the effects of zinc did not account for confounding factors like participants' dietary intake and other dietary components (like magnesium or vitamin B6) and lifestyle factors (like physical activity) [24]. This may affect the accuracy of conclusions regarding zinc’s role in PMS symptom relief. Mechanistic insights reveal that zinc is essential for the synthesis and release of gonadotropins (FSH and LH) by modulating hypothalamic-pituitary-gonadal axis function, thereby influencing ovarian follicle development, hormone secretion, and receptor sensitivity [25].

Sunflower and sesame seeds are included in the luteal phase. While sunflower seeds are rich in vitamin E and selenium, two nutrients that support progesterone and have an antioxidant effect, sesame seeds offer lignans and phytoestrogens to balance the level of estrogen. Vitamin E plays a vital role in enhancing corpus luteum function by stimulating progesterone synthesis, which is essential for maintaining the luteal phase and preparing the endometrium for potential pregnancy. Additionally, its antioxidant properties protect the corpus luteum cells from oxidative damage, supporting their viability and function. This dual action helps sustain hormonal balance and promotes reproductive health. Vitamin E has been associated with increased progesterone production, and selenium protects against oxidative stress and supports thyroid function, both of which are essential for hormonal balance [26]. The antioxidant properties of these micronutrients reduce oxidative stress, a known disruptor of endocrine function, thereby contributing to improved hormonal homeostasis [27].

Many studies investigating micronutrient supplementation tend to evaluate isolated nutrients rather than the complex combinations typically found in whole foods such as seeds, which contain a range of bioactive compounds including zinc, omega-3 fatty acids, and lignans. This approach limits the ability to determine whether observed effects are attributable to a single nutrient or the synergistic interaction of multiple components. Moreover, the relatively short duration of some trials, often limited to three months, may not be sufficient to capture potential long-term benefits. Furthermore, the findings' ability to be applied broadly is limited by the absence of diverse participant representation, such as women with various menstrual disorders.

Additionally, the fiber in these seeds promotes healthy digestion, which is necessary for regular hormone secretion and metabolism. The gut microbiota plays a crucial role in estrogen recirculation through the enterohepatic pathway, where certain gut bacteria produce enzymes such as beta-glucuronidase that deconjugate estrogen metabolites excreted in bile, allowing free estrogen to be reabsorbed into the bloodstream. This process helps maintain optimal circulating estrogen levels and supports hormonal balance. Dysbiosis or imbalance in gut bacteria can disrupt this cycle, potentially leading to estrogen-related disorders such as PMS and menstrual irregularities [28]. A summary of the benefits of the seeds in the menstrual cycle is presented in Table 4. In general, aligning menstrual cycle phases with the targeted nutritional intake of nutrient-rich seeds may support hormonal balance and help alleviate symptoms associated with premenstrual syndrome (PMS) and menstrual irregularities [11]. 

**Table 4:** Summary of Nutritional constituents in seeds and their impact on the menstrual cycle Data in this table are drawn from the following studies included in this review: Ref [10], Ref [20], Ref [21], Ref [22], Ref [23], Ref [24], Ref [25], Ref [26], Ref [27], Ref [28]

Seed Name	Compound present	Research Finding
Flax Seeds	Lignans	Help improve ovulatory function which in turn helps to regulate menstrual cycle
Sesame Seed	Lignans	Counterbalance the level of estrogen
	Phytoestrogens	Counterbalance the level of estrogen
Pumpkin Seed	Zinc	Help produce FSH and LH which help induce ovulation
Sunflower Seed	Vitamin E	Increase production of progesterone
	Selenium	Aids thyroid health and is an oxidative protector – crucial for hormone balance

Limitations

The findings of this review should be interpreted in light of several limitations. Most included studies were geographically clustered in South Asia and the Middle East, which may limit the generalizability of results to other populations. Discrepancies in outcomes, such as improvements in follicle-stimulating hormone (FSH) levels without corresponding changes in hirsutism, may be explained by variations in intervention duration, baseline symptom severity, and participant heterogeneity in age, BMI, metabolic status, and lifestyle factors. Some studies monitored patient compliance through regular phone calls, text messages, and by requiring patients to visit the clinic to consume the powder. While other studies provided only instructions and later assessed hormonal levels to evaluate any improvements. Regardless, adherence to seed intake protocols was not objectively verified, and none of the studies evaluated potential interactions with concurrent medications or hormonal therapies. Furthermore, variability in study design, outcome measures, and follow-up periods underscores the need for standardized protocols and longer-term studies to better clarify the relationship between hormonal modulation and clinical symptom improvement.

Future Scope of Research

Although the study’s results are favourable, several crucial areas require further exploration to better understand the therapeutic potential of seed cycling in PCOS and PMS. Larger, multicenter randomized controlled trials using double-blind, placebo-controlled designs are needed to strengthen the validity of findings. Standardization of seed type, dosage, and timing will facilitate more meaningful comparisons across studies. Further research into the underlying biological mechanisms will enhance comprehension of physiological effects. In particular, future research should investigate the interaction of seed-derived phytoestrogens with estrogen receptors, the modulation of the hypothalamic-pituitary-gonadal axis, and the antioxidant effects on hormonal regulation. Comparative studies with other dietary and lifestyle interventions could clarify seed cycling’s relative effectiveness within broader treatment frameworks. Additionally, incorporating assessments of psychosocial outcomes such as mood, stress, and quality of life, especially in PMS patients, may provide a more comprehensive evaluation of benefits. Finally, integration of digital health tools and cost-effectiveness analyses could support clinical and public health decision-making.

## Conclusions

This systematic review suggests that seed cycling could be a positive complementary approach for managing the symptoms of PCOS and PMS. Improvements in lipid metabolism, insulin resistance, menstrual regularity, and hormonal profiles (including FSH, LH, and prolactin) were noted in the included studies, especially in PCOS patients. In addition, seed cycling contributed to overall symptom relief by alleviating dysmenorrhea and reducing PMS-related discomfort. There is conflicting data, however, about how it affects glycemic control, skin changes, and hirsutism. The current body of evidence is constrained by small sample sizes, variation in intervention protocols, and a lack of long-term follow-up, despite promising findings. Hence, well-designed, large-scale randomized controlled trials are warranted to confirm the efficacy, safety, and mechanisms of seed cycling as a therapeutic option in hormonal regulation and menstrual health.
